# Advances in the Application of Infrared in Food Processing for Improved Food Quality and Microbial Inactivation

**DOI:** 10.3390/foods13244001

**Published:** 2024-12-11

**Authors:** Christian K. Anumudu, Helen Onyeaka, Chiemerie T. Ekwueme, Abarasi Hart, Folayemi Isaac-Bamgboye, Taghi Miri

**Affiliations:** School of Chemical Engineering, University of Birmingham, Birmingham B15 2TT, UK; h.onyeaka@bham.ac.uk (H.O.); theresaekwueme0622@gmail.com (C.T.E.); a.hart@bham.ac.uk (A.H.); f.j.isaac-bamgboye@bham.ac.uk (F.I.-B.); t.miri@bham.ac.uk (T.M.)

**Keywords:** infrared heating, food processing, microbial inactivation, food quality, food safety, thermal treatment

## Abstract

Food processing is a fundamental requirement for extending the shelf life of food products, but it often involves heat treatment, which can compromise organoleptic quality while improving food safety. Infrared (IR) radiation has emerged as a transformative technology in food processing, offering a rapid, energy-efficient method for inactivating microbial cells and spores while preserving the nutritional and sensory attributes of food. Unlike traditional heating methods, IR technology enhances heating homogeneity, shortens processing time, and reduces energy consumption, making it an environmentally friendly alternative. Additionally, IR processing minimizes water usage, prevents undesirable solute migration, and maintains product quality, as evidenced by its effectiveness in applications ranging from drying fruits and vegetables to decontaminating meat and grains. The advantages of IR heating, including its precise and even heat diffusion, ability to retain color and nutrient content, and capacity to improve the microbial safety of food, position it as a promising tool in modern food preservation. Nevertheless, there are gaps in knowledge with respect to optimal application of IR in foods, especially in the maintenance product quality and the impact of factors such as IR power level, temperature, wavelength (λ), food depth, and target microorganisms on the applicability of this novel technology in food systems. Recent research has attempted to address challenges to the application of IR in food processing such as its limited penetration depth and the potential for surface burns due to high energy which has delayed the widespread utilization of this technology in food processing. Thus, this review critically evaluates the application of IR in food safety and quality, focusing on factors that affect its effectiveness and its use to moderate food quality and safety while comparing its advantages/disadvantages over traditional thermal processing methods.

## 1. Introduction

Food is vital for the maintenance of humans and animals. Foods undergo processing and preservation to improve their quality, extend shelf life, and prevent the proliferation of microbial agents, which can result in spoilage and foodborne illnesses [1]. Various technologies are employed for the maintenance of the freshness of product characteristics while improving shelf life, safety, and nutrition [2]. Because of the ever-changing nature of food products and longer food supply chains, there are increasing risks associated with foods [3]. This is mainly from the activities of foodborne pathogens (bacteria, viruses, and parasites), of which the list of microorganisms responsible for foodborne diseases has continued to grow over the years, expanding from toxigenic bacteria such as *Bacillus cereus* to more common agents such as *Salmonella* spp., *Shigella* spp., *Clostridium botulinum*, and *Staphylococcus aureus* and, more recently, zoonotic food-borne bacterial agents [3,4]. Thus, there is a need for the continual development of more fit-for-purpose food processing technologies to meet the needs of the food industries. It was estimated by the WHO that in 2010, there were 600 million cases of foodborne illness, resulting in 420,000 deaths and 33 million disability-adjusted life years (DALYs) globally, with its attendant economic consequences [5]. It is important to note that 30% of this foodborne disease burden is on children under the age of five (5) [6]. Of particular interest in food processing technology development are bacteria spores (more commonly, spores of *Bacillus* and *Clostridium*), because of their recalcitrant nature and difficulties associated with their removal from foods and food processing surfaces. Furthermore, they are extremely resistant to thermal and non-thermal food processes [7] and are key players in major food borne illnesses outbreaks worldwide. In view of the problems caused by microbial cells/spores and increased consumer demand for tasty, nutritious, natural, and easy-to-handle food products, the food industry is increasingly investigating the replacement of traditional food preservation techniques (intense heat treatments, salting, acidification, drying, and chemical preservation) with new preservation techniques [8]. Food processing technologies employ a variety of techniques to inactivate microorganisms and improve quality and stability. Many researchers have investigated alternative food processing and preservation methods and their effectiveness against relevant pathogens and spores for various food items due to the need to improve food safety and extend shelf life.

Heat is used in traditional food processing to inactivate foodborne pathogens. It is an effective way to treat a variety of foods and depends on the transfer of heat by conduction, convection, and radiation [9]. Heat can be applied in various formats, including long-time, low-temperature, or short-time high-temperature applications. Some traditional heat treatments include hot smoking and sun drying at 71 °C and 31 °C, respectively, especially for seafood such as shrimps and fishes to dehydrate, reduce microbial load, and improve shelf-life and quality of food products [10]. Other high-temperature processes such as roasting and baking have been used in traditional food processing to ensure long shelf life and food safety. However, traditional thermal processes suffer from heat transfer limitations, with a gradient of temperature exposure from the outside to the inside of the food, which can possibly result in over-processing, causing severe damage to the sensory, nutritional, and functional properties of foods [11]. In addition, some foods cannot withstand traditional high temperature heat treatments and potentially create undesirable outcomes. Foods such as raw oysters, mussels, and clams pose a risk of bacterial or viral foodborne disease, as heat treatment is either undesirable or impossible to use [12,13]. Importantly, consumers over the years have shown preference for minimally processed foods that are safe, free from microbial agents, and with a long shelf life. These are difficult to achieve using the current conventional heat treatment, thus necessitating a shift in heat application technology towards methods such as infrared (IR) heating [12].

IR radiation, also known as IR light, is a type of radiant energy that is invisible to humans but can be felt as heat. All objects in the universe emit some level of IR radiation, but the sun and fire are two of the most obvious sources [14]. Thus, IR is one of the oldest heat treatment techniques for food. The IR region of the electromagnetic spectrum lies between visible light and microwaves, with wavelengths (λ) ranging from 0.5 to 100 µm. 

This review aims to elaborate on the application of IR heating for food processing and the prevention of microbial contamination of food. It also discusses the interaction of IR with food materials and provides an up-to-date perspective on future research within this field. The review covers the concept of IR heating, microbial contamination of foods, the application of IR in the inactivation of microbial cells and spores in foods, and the advantages and disadvantages of IR processing of foods.

## 2. The Electromagnetic Spectrum and Concept of Infrared Heating

The term ‘electromagnetic (EM) spectrum’ is used to describe the entire range of light that exists. Most of the light in the universe is invisible to us, from radio waves to gamma (ϒ) rays [15]. In a vacuum, all EM waves travel at the speed of light, but at a variety of frequencies, wavelengths, and photon energies. The EM spectrum encompasses all radiation and is divided into numerous sub-ranges, or portions, such as visible light and ultraviolet (UV) radiation [16]. Visible light is the most common type of EM radiation that can be detected with the unaided eye [17]. The visible light spectrum oscillates between 400 and 790 terahertz (THz), with varying colors based on their wavelength and frequency. The longest visible wavelength is interpreted as red, while the shortest is seen as violet. The visible spectrum represents only a small portion of this vast range of energies on the EM spectrum; there is an enormous assortment of light that is not visible [15]. The other types of electromagnetic radiation as we move down in frequency from red light include IR, microwaves, and radio waves. All these types of radiation are invisible to the eyes and have less energy than visible light. As light frequency progresses from purple, the invisible spectrum including UV radiation, X-rays, and gamma rays (ϒ) are encountered, with these forms of radiation having higher energies than visible light. 

As previously highlighted, IR radiation is a type of radiation that exists between the visible and microwave regions of the EM spectrum. This means that the energy associated with IR radiation is less than that of visible light but greater than that of microwaves. IR radiations as a result of having the energy to initiate molecular vibrations act by inducing recurring oscillations of the positions of atoms around their bonds, while the entire molecule is in constant translational and rotational movement [18].

### 2.1. The Infrared Spectrum

**Figure 1:**
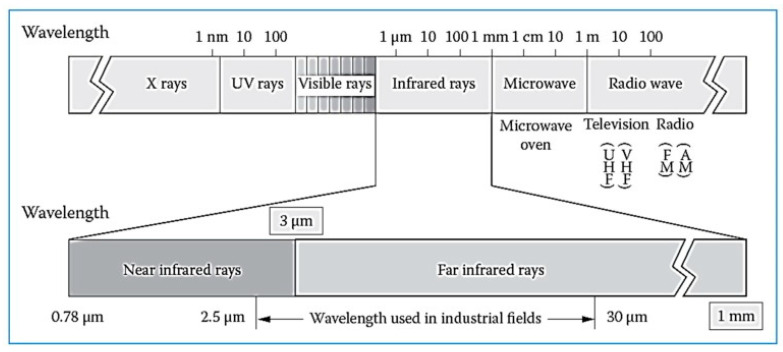
Electromagnetic spectrum and the infrared ranges [20].

Different wavelengths have been given by different authors as the starting point of IR radiation within the electromagnetic spectrum. IR radiation has been given a wavelength range of 0.75 to 1000 µm [19] and 0.76 to 1000 µm [20], while Tsai et al. [21] gave a range of 0.78 to 1000 µm and further divided IR into three distinct zones: near-infrared (NIR) with a wavelength of 0.78–3.0 μm, mid-infrared (MIR, 3.0–50.0 μm), and far-infrared (FIR, 50.0–1000.0 μm). Figure 1 shows the Electromagnetic spectrum and IR wavelength ranges. Other classifications of the IR spectrum exist, with the division of the IR spectrum into five regions becoming the most predominant scheme. These regions include near-infrared (NIR), short-wavelength infrared (SWIR), mid-wavelength infrared (MWIR), long-wavelength infrared (LWIR), and far-infrared (FIR) [19] as follows:

- Near-infrared (NIR)–This is the region that is close to visible red with a wavelength between 0.75 and 1.4 µm. This region has gained vast applications in fiber-optic communication, night vision devices, remote controls, astronomy, remote monitoring, material science, the medical field, and agriculture.
- Short-wavelength infrared (SWIR)–This ranges from 1.4 to 3 µm and is used for long-distance communications. It is also used in short-wave infrared imaging (SWIR) cameras and night vision goggles, both of which are important in the military.
- Mid-wavelength infrared (MWIR)–This ranges from 3 to 8 µm and is used in guided missile technology, IR spectroscopy, communication, and the chemical industry.
- Long-wavelength infrared (LWIR)–This is the thermal infrared region, which is used to detect thermal emissions that do not require additional illumination (thermal imaging). This is applied extensively in astronomical telescopes and fiber optics. This division has a wavelength between 8 and 15 µm.
- Far-infrared (FIR)–This ranges from 15 to 1000 µm and is used in IR lasers, in astronomy, in IR saunas, and in the medical field.

### 2.2. Characteristics of Infrared and Its Application in Heating

IR heaters operate by converting electricity into heat waves by radiating IR into an enclosed space. The material within the space, such as food, gets heated ostensibly through the vibration of water molecules at high frequencies at 60,000 to 150,000 MHz. Thus, they operate without contact with the medium to be heated. IR heating technology is distinguished by heating homogeneity with a high heat transfer rate, a short heating time, low energy consumption, and improved product quality and food safety [22]. The reduction in energy consumption in IR heaters makes them more energy efficient and environmentally friendlier than conventional heaters [20,23]. With the recent development of small and compact IR radiation equipment with high accuracy in controlling factors, there is a reduction in the high energy demand and costs of such equipment [24]. When these newer and more compact IR radiation technologies are complemented with other technology, there is a noted increase in efficiency and energy savings as exemplified by a study by Hebbar et al. [25], which combined infrared with convective drying for vegetables, resulting in a 48% reduction in drying time and a reduction in energy consumption by 63%. Overall, IR radiation has high thermal efficiency and is therefore regarded as a valuable energy source for food applications [20]. Although empirical studies show that IR radiation is advantageous over conventional heating, including having shorter heating times; higher heat transfer coefficients; lower quality losses; uniform heating; versatile, simple, and compact equipment; and the ability to save energy under similar conditions [2], questions have been raised about the efficacy of IR radiation in food processing, especially with regard to its ability to penetrate food materials and inactivate microbial cells and spores. 

The depth to which EM radiation can penetrate materials is referred to as its penetration depth. EM waves can travel very far into a material and be reflected or absorbed at the surface, depending on the material’s nature [26]. The penetration depth for a given material can vary for different EM waves and is usually not fixed [27]. IR radiation has a relatively wide wavelength range (780–1400 nm), and this impacts its depth of penetration in foods [28]. Recent studies show that many materials are capable of absorbing IR at the surface and not deep within the food matrix, resulting in an uneven heating of relatively thicker food materials. Since the effects of infrared radiation on microbial inactivation diminish with sample thickness, understanding penetration depth is crucial for designing an efficient IR processing system and for surface pasteurization procedures. The absorptivity of IR radiation by the food product is a significant factor. The thickness of the food product, the water activity, the state of the food product (i.e., solid, liquid, powder, etc.), the composition, and the infrared radiation wavelength are all factors that determine how deep an IR penetration occurs [29,30]. Additionally, a food product’s chemical composition, physical characteristics (i.e., density, porosity, moisture content, etc.), and physico-chemical condition are crucial to the penetration depth, which is a critical parameter in the design of industrial IR sterilization processes for food products [30]. Dong et al. [31] evaluated the penetration of NIR diffuse reflectance light on a variety of fruit peels. Results showed ease of NIR penetration on thin-peel fruit relative to thick-peel fruit like citrus, which shows the relationship between the thickness and composition of food material with the penetration depth, with LWNIR being mostly unsuitable compared to SWIR due to noise interference and spectral distortion. Again, the results obtained by the study of Eliasson et al. [32] comparing the IR and microwave heating penetration depth on paprika powder support the report of Dong et al. [31], indicating a limited penetration depth by IR, which makes it better suited for surface heating technology. The experimental setup required a relatively longer heating time for IR, which doubles the time required for heating completion of microwave heating due to the sample thickness and a lower penetration depth. Several other studies have reported similar penetration depth of IR, such as in relation to grain bed depth [33], fruit and vegetable peeling [34], and roasting nuts [35].

Additionally, food materials with high moisture content have the potential to absorb IR energy quickly, which favors outermost heating only, thus making the technology more suitable for dryer foods [36]. IR heating is commonly best adapted for applications requiring rapid surface heating rather than deep penetration. Today, this limitation of penetration depth has consistently been improved by adopting combinatorial approaches with the use of IR heating, for example, contact ultrasound and IR radiation combination during hot air drying of air-dried beef [37], VIS-NIR of starchy and protein foods [38], far-infrared radiation heating-assisted pulsed vacuum drying (FIR-PVD) of blueberries [39], contact ultrasound and IR radiation drying of taro slices [40], combined medium- and short-wave IR and hot air drying (HAD) of sponge gourd slices, and intermittent combination of IR and HAD of sweet potatoes [41]. 

### 2.3. Sources of Infrared Radiation in Food Processing

IR radiation is emitted by any substance that is above absolute zero in temperature. Most sources of light and heat, including the sun, emit some energy in the IR range [20]. Industrially, electric heaters and gas-fired heaters/burners are two of the most common types of IR radiators used for IR heating processes. IR radiation produces temperatures in the range of 343–1100 °C for gas heaters and burners and 1100–2200 °C for electric heaters [19]. Electrical IR emitters are made up of a metal filament such as nichrome wire, iron-chromium wire, and tungsten filament enclosed in a sealed container filled with inert gas or left empty. IR radiation is produced when an electric current is passed through this high-resistance wire. Although gas heaters/burners are expensive, their operating costs are low when compared to electric heaters that emit IR radiation. This is due to their ease of control, fast heating rate, and clean energy. However, IR electric heaters are more widely used than gaseous heaters due to initial cost and because the electric IR emitters are more adaptable when it comes to producing the wavelength needed for a specific application. Depending on the voltage applied to the emitters, IR radiators can emit wavelengths ranging from short to long IR wavelengths, whereas gas heaters and burners emit medium to long IR wavelengths [42]. Traditionally, with regard to efficiency, electric IR heaters have efficiencies ranging from 40% to 70%, whereas IR gas heaters have efficiencies ranging from 30% to 50% [43]. However, more recent studies have recorded efficiency as high as 85% [44], with efficiency not only dependent on the type of heater but on several other factors including reflectors, heating medium, air velocity, and, in the case of electric heaters, the type of filament [24,44]. 

## 3. Application of Infrared Radiation in Foods

IR energy does not depend on air and is transformed to heat when it is absorbed by the material being heated. Because air and gases absorb very little IR energy, the IR heating process allows for effective heat transmission without the need for direct contact between the heat source and the substance to be heated. Foods with a high water content absorb MIR and FIR energy most effectively through various modes of vibration, resulting in the radiative heating process [45]. Vibrations can change the bond’s length (stretching) or angle (bending). Some bonds are symmetrical, while others are asymmetric [46]. When IR radiation is used to heat or dry moist materials, it impinges on the exposed material and penetrates it, converting the radiation energy into heat. It can penetrate deeply into food products with product-to-emitter gap sizes (PEG) of up to 110 mm and an intensity of 15.71 kW/m^2^ at a wavelength of 3.2 µm, dependent on surface moisture and temperature [45].

Due to the inherent advantages of IR radiation over conventional heating systems, its application in food processing is gaining momentum. Various food products have been dried, baked, roasted, blanched, pasteurized, and sterilized using IR heat [42].

### 3.1. Drying and Dehydration

Drying is an extremely important way to preserve and maintain food in the food industry. Drying focuses on the reduction in the water content of a food by evaporation for culinary or preservative purposes [47]. This is different from thermal treatment, which focuses on using very high temperatures to achieve a broader goal such as sterilization or altering the composition of a food at temperatures higher than that employed for drying and does not necessarily remove water from a food [48,49]. Conventional drying technologies commonly used to dry food products include hot air, freeze-drying, and microwave drying. There are advantages and disadvantages to each of these methods. Hot air drying is the most commonly used technique to preserve food via drying, but it is not energy efficient, and the food product’s quality could decline over time, making it less competitive. Drying with a microwave, on the other hand, can easily lead to overheating and degradation of food quality. Freeze-drying has a constrained use and is typically used to dry temperature-sensitive and high-value food products due to its comparatively long drying time and high drying cost. Utilizing IR radiation to supply heat is an alternative drying process that is gaining prominence for several agricultural products. Compared to other common drying methods, IR drying offers many advantages, such as high-quality products, higher energy savings, efficiency, and a higher thermal transfer rate. As IR radiation is absorbed by water molecules within the food product, it causes them to vibrate and heat up internally, which drives moisture radially outward to the external surface. The impact of a combination of various infrared drying approaches (e.g., IR, IR-hot air, IR-vacuum, IR-fluidized bed, and IR-microwave) on the drying time, effective moisture diffusivity, and textural and quality attributes of dried apple, quince, grapefruit, lemon, persimmon, banana, peach, mushroom, carrot, pumpkin, garlic, and onion has been reviewed in a published article [50]. Compared with conventional drying methods, IR drying offers 51.1% less energy consumption, short drying time, uniform heating during drying, and excellent quality food products [36]. IR drying is also effective for high-value food products. Based on experimental study results, ginseng dried more quickly using an IR dryer. At 40 °C, 50 °C, and 60 °C, the drying time of ginseng under FIR was reduced by 33.3%, 25%, and 20%, suggesting that FIR has a higher drying efficiency, and this improvement becomes more noticeable as the temperature decreases [51]. In addition, it was found that IR drying was significantly more energy-efficient than hot air drying, with the specific energy consumption decreased by up to 40% under far IR. Importantly, because the aim of drying is to reduce the moisture content of a food, it is important to avoid using very high IR power, intensity, and drying temperatures, as these will cause the food product to overheat [36], becoming more of a thermal treatment than drying and possibly affecting the organoleptic and physicochemical properties of a food. 

In addition to direct IR drying, another approach used in the drying of food is convection-assisted IR drying. De Souza et al. [52] combined z convection current of unheated air with IR to dry bananas, eliminating the risk of damaging the food by increased heating associated with using IR alone. Vacuum IR drying involves the use of a closed environment, which works by decreasing the boiling point temperature and hence reducing drying temperature [53]. A study [37] utilized this drying method in drying cistanche slices through FIR vacuum drying to study the effect of an ultrasound (US) pre-treatment on the process at different times. Overall, high-quality dried materials were obtained in a short time. Again, [54] employed moving bed systems in the drying of ginger with microwave-IR radiation. The microwave infrared vibrating bed drying (MIVBD) equipment ensures the distribution of the EM field by altering the position of materials to achieve drying uniformity and maximal absorption of radiation, thereby improving the quality of dried products. Batch and continuous IR drying systems are also adopted for the drying of food products relative to the quantity of the materials being dried [55]. Other methods of IR drying may involve thickness control, representing a diffusion-controlled drying mechanism adopted to reduce shrinkage problems commonly associated with air drying [56].

A study [57] used short- and medium-wave IR to improve the drying properties and color of apple slices while also shortening the drying time. Another study, [58], examined IR heating practicability to improve banana drying rate, assessed dried product quality, and identified models for drying prediction characteristics. Results indicate that significant humidity reduction and increased drying rates were achieved with IR drying, compared to conventional heat drying. Their findings showed that inactivation of the enzyme occurred faster in IR than in hot air drying.

### 3.2. Other Infrared Applications in Food Processing 

IR heating has also been shown to be useful in various other food processing applications, including roasting, frying, broiling, heating, and cooking meat and meat products, soybeans, cereal grains, cocoa beans, and nuts [59]. Olsson et al. [60] reported that when compared to heating in a conventional household oven, IR radiation and jet impingement increased the rate of color development of the crust and shortened the heating time of parbaked baguettes during post-baking. Furthermore, combining IR and impingement heating resulted in the quickest color development. The rate of water loss increased due to a faster heat transfer rate, but total water loss decreased due to a shorter heating time. This is important in terms of the organoleptic quality of the food and microbial safety. 

IR has also been adopted in peeling, polyphenol recovery, freeze-drying, antioxidant recovery, sterilization, and the manufacture of juices [20]. In the recovery of polyphenol, IR-assisted extraction was employed for the recovery of polyphenols from pomegranate peels, which produced the highest concentration of polyphenol (152 mg/gDM) relative to other methods. In a recent study, IR technology was adopted to assist the optimization of peonidin 3-O-glucoside and cyanidin 3-O-glucoside from purple corn cobs [61]. Similarly, El Kantar et al. [62], reported a comparison between IR-assisted phenol extraction and conventional extraction using a water bath with IR extracts, showing relatively high bioactivity. IR technology has been shown to optimize the yields of antioxidant phytochemicals and pigments for the inactivation of microorganisms. Calleja-Gomez et al. [63] reported an optimization of antioxidant and phenolic activity with a simultaneous recovery of chlorophyll and carotenoids, showing that IR enhanced recovery by 10.06%. 

IR technology has also been adopted in the peeling of fruits and vegetables without the use of water and chemicals, which ensures an environmentally friendly peeling technology. Li et al. [64] reported a puncture technique in the peeling of walnuts, which involves the stimulation of the internal pressure of the kernel surrounding the flesh of the fruit during IR heat radiation. Following this, the cell collapses the surface of the shallow cross-section of the walnut kernels, likely due to the low penetration depth of IR radiation causing sudden heating of the shallow cells, leading to rupture of the oil body membrane and consequent oil leakage. This brings about cell contraction, which results in peeling while causing only superficial cell damage to walnut kernels. This approach minimizes internal damage and maximizes kernel quality. The peeling of several other fruits using IR technology has been reported with fruits such as tomatoes [65], hazelnuts [66], kiwi [67], and ginger [68]. 

IR has also shown great potential in freeze-drying of food products. The efficacy of IR-assisted freeze-drying (IRFD) was studied to produce banana snacks. Khampakol et al. [69] installed an IR lamp in a freeze-dryer. Continuous freeze-drying with IR significantly reduced drying time by over 70%, thereby consuming less energy. The potential of IR in freeze-drying has also been reported in sweet potatoes [70], aloe vera, strawberries [71], bananas [72], and apples [24]. Similarly, in the manufacture of juices, IR is broadly employed due to the potential of IR in quality optimization [73]. Analytically, IR has shown potential in being used as a predictor of sugar content of fruit juices [74], profiling of nutritional content in juices using FTIR [75], classification and characterization of fruit juices for a product-customized flash pasteurization [76], and the differentiation of commercial juices of the same fruit to evaluate quality of commercially marketed juices [77].

### 3.3. Inactivation of Enzymes

It is commonly desirable to inactivate enzymes during food processing to maintain the nutritional quality, texture, color, and flavor of foods. To increase shelf-life stability or achieve desired product quality, food products may need to be blanched first using steam or hot water to inactivate enzymes and then dried with heated air to reduce water activity [78]. This is because the activities of enzymes may result in food quality declines during storage, particularly for fruits and vegetables. The use of conventional thermal processing for enzyme inactivation with its associated low energy efficiency and long processing time results in the loss of the nutritional and organoleptic qualities of food products leading to quality deterioration. IR heating can be used to inactivate enzymes effectively. The presence of enzymes in either raw or processed plant-based foods potentially catalyzes reactions within food that reduce food quality and accelerate spoilage. The inactivation of these enzymes is important in reducing detrimental effects encountered during food processing and preserving the food organoleptic properties. Polyphenoloxidase (PPO), peroxidase (POD), pectinase, pectinesterase, lipoxygenase, alkaline phosphatase, and beta-glucosidase are common enzymes implicated in food spoilage [79]. For example, PPO and POD causes color changes in sugarcane [80]. Enzyme inactivation is achieved by the structural alteration or destruction of bonds of the tertiary protein structure and hydrogen bonds of the secondary protein structure of the enzymes. The mechanism by which IR inactivates enzymes is mainly through thermal damage of the enzyme active sites and unfolding the enzyme structure [20]. Nevertheless, certain factors may affect the inactivation of enzymes, such as the product parameters and temperature. For example, PPO inhibition in potatoes requires a temperature of 65 °C within the product. Enzymatic inactivation will require an increase in IR intensity and a reduction in product thickness to enhance penetration depth [20]. Inactivation of other enzymes such as those involving action of lipases and α amylases requires a bulk temperature of 30 to 40 °C [2].

Exposure of enzymes to short IR treatments can bring about significant structural change and inactivation of the enzymes. The activity of many enzymes in foods can result in oxidation and the production of off-flavor and off-color. Thus, the inactivation of these enzymes can directly result in the extension of the shelf life of food and improved food quality. Exposing lipoxygenase, an enzyme that causes soybean deterioration, to 60 s of IR treatment resulted in 95.5% inactivation [19]. This confirms that enzymes and their activities can be rendered inactive by infrared (IR) heating, and this can be achieved with little or no impact on the food product’s nutritional qualities. Similarly, [81] reported a significant decrease in lipoxygenase activity of chickpea and green lentil flours when treated with IR heating (130 and 150 °C). Similarly, IR radiation affects enzymatic reactions involving lipases and amylases. It has been highlighted that lipase activity was reduced by 60% when exposed to FIR radiation for 6 minutes and by 70% when exposed to thermal conduction [19,82]. This shows that there is a potential to use IR heat treatment to effectively inactivate enzymes, which can cause undesirable organoleptic changes in foods to improve food quality and safety.

The inactivation of the enzymes α-amylase and lipase during the far-infrared (FIR) radiative heating approach has been studied and contrasted with activity changes due to thermal conduction heating [82]. As long as the temperature profiles of the enzyme solutions remained the same during heating, it was found that the decline in enzyme activity for the two methodologies was greatly comparable. Comparing the inactivation of α-amylase with bacterial death (*Escherichia coli*) using FIR and conductive heating suggests that FIR heating may be able to maintain enzyme activity levels while achieving a specific pasteurization target level at lower temperatures than conductive heating. The results prove that in the utilization of FIR heating, the death rate constant of *Escherichia coli* is larger than when using conductive heating at all temperatures investigated. This suggests that FIR heating may be more effective than conductive heating for pasteurization. In another study, the effects of baking, far-infrared heating, frying, microwave heating, and steaming thermal stabilization treatments on the enzymes, lipids, and aroma profiles of highland barley flour (HBF) were investigated and reported [83]. Based on the results, the HBF lipase activities in IR, baked, microwaved, fried, and steamed treated highland barley flour were significantly decreased by 30.2%, 13.2%, 25.8%, 40.9%, and 25.8%, respectively. This implies that except for frying, IR heating can significantly inactivate lipase, catalase, and lipoxygenase activity. Compared to IR heating, a large amount of energy is required for frying, baking, and steaming. To gain insight into the inactivation of the enzymes polyphenol oxidase (PPO) and ascorbic acid oxidase (AAO) and to understand how it affects color change and the retention of vitamin C and β-carotene, a study compared the effects of IR dry blanching with traditional water blanching at 90 °C for 2 min and 65 °C for 10 min before hot air-drying mangoes [84]. Both water and IR blanching methods at 90 °C for 2 min and 70 °C for 10 min completely inactivated PPO in mangos, whereas AAO showed very little residual activity at the lower temperature. When compared to water-blanched mangos, IR-blanched mangos retained more water-soluble vitamin C and less all-trans-β-carotene. The effects of radiation intensity, slice thickness, and processing time on apple slices exposed to IR radiation heating in a continuous heating mode for attaining concurrent infrared dry-blanching and dehydration were investigated and evaluated based on the heating and drying rates, product temperature, moisture reduction, residual polyphenol oxidase (PPO) and peroxidase (POD) activities, and surface color change [78]. Compared to low radiation intensity and/or thick slices, high radiation intensity and/or thin slices caused the product temperature to rise more rapidly, removed moisture more quickly, and inactivated PPO and POD more swiftly. These findings suggest that combining infrared radiation heating with microwaves and other widely used conductive and convective heating methods has enormous promise for achieving energy efficiency and practical usability in the food processing sector. However, a cost-benefit analysis needs to be conducted.

### 3.4. Microbial Contamination of Foods and IR Pathogen Inactivation

Food remains vulnerable to spoilage and pathogenic microorganism contamination throughout the manufacturing chain, retail stores, restaurants, and consumers’ homes [85]. The damage caused by spoilage and contaminated food affects both the food industry (economic loss, reputational damage, and punishment under local law) and consumers in terms of foodborne illnesses and financial loss [86]. Food spoilage caused by microbial activity involves numerous complex mechanisms that negatively impact the organoleptic quality of foods, resulting in their rejection by consumers. Bacteria and fungi are two major groups of microorganisms that have been linked to food spoilage [87]. Some pathogenic bacteria, such as *Clostridium botulinum*, *Clostridium perfringens*, *Bacillus subtilis*, and *Bacillus cereus*, are capable of spore formation and thus are extremely resistant to heat [88]. Some of them can produce heat-resistant toxins (e.g., *Staphylococcus aureus*, *Clostridium botulinum*), which are a leading cause of foodborne illnesses worldwide. The majority of pathogens are mesophilic, with optimal growth temperatures ranging from 20 to 45 °C, which are common storage temperatures for most foods. Certain foodborne pathogens (psychotropic), such as *Listeria monocytogenes* and *Yersinia enterocolitica*, can grow in refrigerated environments or at temperatures below 10 °C [88]. These organisms have been found in commercial freezers used for food preservation [89] and can pose a significant hazard to food safety. Similarly, viruses and fungi play an important role in foodborne illness and food spoilage. To inactivate microorganisms in food, a variety of methods are used, either individually or in combination. In addition to conventional approaches for the control of microbial agents such as high and low temperature control, pH moderation, and use of organic acids and antimicrobial preservatives [90], IR can be employed to inhibit microbial agents such as vegetative and spore forms of bacteria, yeasts, and molds in solid and liquid foods [59]. IR acts by altering the DNA, RNA, cell wall, proteins, and ribosomal units of target organisms. The effectiveness of IR heating in microbial inactivation is determined by IR wavelength and bandwidth of the radiation source. Other factors include; temperature, thickness, moisture content, depth and chemical matrix of the food sample, physiological phase of the microorganisms, and type of targeted microorganisms [59]. As the power of the IR heating source increases, so does the total energy absorbed by microorganisms, resulting in microbial inactivation. Studies have highlighted that IR heating improved fungal inactivation [91]. A study [92] used optical bandpass filters and selective IR heating in the wavelength range of 5.88 to 6.66 µm to inactivate *Aspergillus niger* and *Fusarium proliferatum* in corn meal. The chosen wavelength denatures the protein in microorganisms, resulting in a 40% increase in inactivation of *A. niger* and *F. proliferatum* when compared to standard IR heating.

## 4. Thermal Death Kinetics Model

Various food processing treatments have been widely adopted in the food industry for the destruction of spoilage and pathogenic microorganisms. Heat treatment has been specifically adopted for the loss of viability of vegetative bacteria (pasteurization) and long-term safety of food during storage by inactivating microbial spores (sterilization) [93]. Traditional exponential inactivation kinetics based on thermodynamics predicts that at constant temperature, thermal death of bacteria is exponential to time. Heat resistance is expressed in terms of exposure time needed to inactivate 90% of a bacterial population by Log^10^ cycle at a constant temperature based on a target structure involved in cell inactivation like DNA [94], and various thermal processes in food industries have based food processing techniques on these thermal inactivation kinetics. These treatment processes vary widely due to the varying treatment requirement of microorganisms and variability among [95,96]. Additionally, evidence that supports damage of cell structures capable of solely causing cell death has emerged, as cell death based on DNA inactivation will require enormous heat that may have caused cell death prior to DNA inactivation in vegetative cells. This has led to non-logarithmic thermal death kinetics curves based on increasing severity of cell injury above a threshold, microbial adaptation, existence of a non-single target inactivation, and certain spores that behave differently with respect to heat resistance and has deviated thermal death kinetics from the traditional standpoint [93].

Eradication of microorganisms is dependent on varying factors and has in recent times launched various models and applauded the use of various techniques in the food processing industry. Of particular interest is the integrated model that combined thermal death kinetics with an IR heat transfer model to predict the survival of fungal spores based on temperature prediction [92]. Individual fungal spores were found to be more lethal when subjected to selective infrared heating. Denaturation of the protein band as a target spectral region of selective heating may also increase fungal spore lethality. This model validates IR heating processes, as it is based on the temperature requirement to predict the survivors under conditions that differ with time.

Tanaka et al. [97] investigated the suitability of the method for surface decontamination in strawberries by combining Monte Carlo FIR radiation simulations with convection-diffusion airflow and heat transfer simulations. The model was a powerful tool for evaluating complex heating configurations such as radiation, convection, and conduction quickly and comprehensively. The computations were validated against thermographic camera measurements. FIR heating produced more uniform surface heating than air convection heating, with a maximum temperature well below the critical limit of around 50 °C. Several factors, such as system rotation, optimized heating cycles, and different heater geometries, can improve system functionality in terms of heating rate and temperature uniformity. The proposed modeling approach can be used to achieve such a goal comprehensively, and the model should be extended to account for mass transfer and volumetric dissipation of radiation power.

## 5. Effect of Infrared Treatment on the Organoleptic and Physicochemical Parameters of Foods

The key challenge faced by the food industries during food and food products processing is the preservation of the nutritional and physical attributes of fresh produce. The appearance of a food product is one of the first attributes a customer notices about a food and strongly influences purchase decisions. The majority of essential nutrients in foods are depleted due to physical and chemical changes during processing, such as high temperatures, low relative humidity, and enzymatic reactions [73]. When foods are thermally processed, the problem is exacerbated, resulting in changes in the appearance of the food. High-temperature processing of sugary food leads to the formation of browning pigments due to non-enzymatic reactions between sugars, ascorbic acid, and amino acids. This browning reaction results in the degradation of a products taste, color, and overall acceptability [98]. Advances in IR technology are being employed to limit browning reactions in food products. IR radiation with wavelengths ranging from 0.5 to 100 µm has been used to preserve and retain the organoleptic properties of foods, particularly juices [73]. Altemimi et al. [73] pasteurized black lime juice (*Citrus aurantifolia*) using IR at a wavelength of 6 µm and stored it at a single temperature of 62.2 °C in an attempt to retain the juice’s native properties, appearance attribute, aroma, taste, viscosity, color, and acceptability. Additionally, the utilization of minimum heat exposure and the elongation of the minimum storage period of foods by IR makes IR a processing method of choice. 

Furthermore, IR processing reduces processing time and preserves the nutrient content of foods. During IR processing, the food’s surface comes into contact with the radiation and heats up, with the heat gradually diffusing into the product’s inner layers. This reduces food processing time. For example, apples slices required 250 min to dry by convention; however, the use of NIR with a peak wavelength at 1200 nm reduces the time required to dehydrate apple slices to 192 min and 104 min at 53.2 °C and 95.7 °C, respectively [24], and improves consistency of the product. Similarly, a positive effect on color development in food is facilitated by IR processing. Tan et al. [99] reports a desirable color retainment based on the Hunter color parameter at a high drying rate in potatoes and pineapples treated with IR radiation at an average heat flux of 0.13 W/m^2^ and 240 V using a heat pumped dryer fixated with two 230 V, 1000 W IR lamps. In addition, Boudhrioua et al. [100] found that the total phenol content of varieties of olive leaves (Chemlali, Chemchali, Zarrazi, and Chetoui) cultivated in Tunisia and processed with IR heating was increased compared to fresh leaves. This was particularly evident with an observable spike in total phenolic content in Chemlali fresh leaves from 1.38 ± 0.02 to 2.13 ± 0.29 (40 °C) after treatment with IR, thus indicating the potential of this technology to improve the organoleptic quality of foods.

With regard to food quality, IR has several benefits. IR processing preserves vitamins and volatile compounds within foods, resulting in very little flavor losses. In relation to vitamin retention in foods, temperature and heating time have been reported to significantly affect the ascorbic acid content of juice using most heating methods. However, it has been shown that IR heating causes less ascorbic acid degradation, resulting in lower browning pigment formation and color variation compared to the conventional thermal processing, which highlights its benefit in the retention of nutritional values [101]. Furthermore, the use of IR heating helps to reduce the heat degradation of beta-carotene and chlorophyll [2], improving the organoleptic quality of foods. For example, a study [102] applied IR radiation in the manufacturing process of green tea. Following treatment using FIR irradiation at 90 °C for 10 min, which was applied instead of a roasting step, the study reported an increase in the total phenol content of green tea from 475.6 to 811.1 mg g^−1^ and the total flavanol content from 175.7 to 208.7 mg g^−1^. Additionally, epigallocatechin and epigallocatechin gallate contents increased from 57.68 and 9.60 to 89.88 and 16.33 mg g^−1^, respectively. These increases improve the quality of the tea and its flavor components. This report is similar to another study on catechin content and nitrite scavenging activity of green tea upon treatment with FIR irradiation at eight different temperatures between 80 °C through 150 °C. Total phenol and total flavanol contents of green tea increased from 244.7 to 368.5 mg g^−1^ and from 122.0 to 178.7 mg g^−1^, respectively [103].

## 6. Factors Affecting Effectiveness of Infrared Radiation

For the control of microbes in foods, a variety of factors affect the effectiveness of IR treatment. Of particular interest are the IR power, temperature, wavelength of radiation used, food depth, and type of microorganism. These factors are strongly interdependent on each other, and the manipulation of these factors affects the efficiency and applicability of infra-red heat treatment. 

### 6.1. Infrared Energy

The level of IR power affects the drying kinetics and quality of food products receiving IR treatment. Increasing IR power level brings about a decrease in drying time and increased drying rate. Increasing the IR power will lead to an increase in the emission intensity of the IR radiation, resulting in stronger activity and dehydration. In a study on grated carrots, the rehydration capacity of the food samples decreased with an increase in IR power due to the formation of a layer that prevented the uptake of moisture after drying [104]. Another study [105] investigated IR drying of sweet potato slices at power levels of 125, 146, and 167 W using three drying models to evaluate drying kinetics. Results showed an overall decrease in the rehydration capacity of potato slices as IR power increased. In another study, the moisture content of longan decreased within a short time, and the rehydration capacity improved when the IR power was increased [106]. Similarly, the IR power impacts the food quality. Increasing the IR power has been shown to impact food color development [107,108], hardness of the final product [109], and shrinkage in foods such as lemon slices [110]. Thus, depending on the food product, the power of the IR source can be adjusted to fit the desired food processing outcome, but this is interdependent on several other factors, such as the thickness of food.

### 6.2. Temperature 

The temperature emitted by IR sources directly impacts the food processing activity, microbial and enzyme inactivation, and food organoleptic quality. A study [111] reported the effect of varying drying conditions including IR intensities and drying air temperature on strawberries. Results show that increased IR intensity favored drying processes in addition to air velocity and increase in temperature up to 100 °C. Similarly, Si et al. [112] reported a significant effect of increased temperature (70–80 °C) on drying rate and rehydration. However, because of the interdependent nature of these variables, excessively high temperatures can have deleterious consequences on foods. For example, increased temperature can negatively impact food coloration. In a recent study [113], alteration in the quality of orange peel and leaves was observed following treatment at a high temperature (70 °C) for a short period of time. Although this high temperature treatment resulted in a higher drying rate, its impact on quality of samples makes it important to consider the optimum temperature requirement of varying food samples during the drying process to create a balance between drying or dehydration and food quality.

### 6.3. Microorganism Type 

Destruction of bacterial spores in food is largely based on activation of vegetative cells by triggering germination followed by inactivation. Hamanaka et al. [114] investigated the effect of IR radiation on inactivation and injury of *B. subtilis* and *B. pumilus* spores. Results showed that FIR radiation activated spores by heat transfer to trigger germinant receptors (GRs), followed by the death of the vegetative forms. This is in line with a similar study on the inactivation of *B. subtilis* pre-heated at varying water activities using an electric heater, with a resultant inactivation of spores at 0.5–1.0 kW [115]. Recent studies show similar responses of vegetative cells and spores to NIR, with results suggesting multiple possible pathways for DNA damage or oxidation disruption of spores caused by photochemical reactions [116].

### 6.4. Wavelength of Radiation 

The wavelength of the IR source significantly affects the applicability of IR in food processing, with the different regions of the IR spectrum being more suited to different food compositions and affecting drying time of foods. A study [117] reported the drying kinetics and quality of shredded squid in an infrared-assisted convective dryer. The treatment process at the wavelength of 2.5–3.0 µm was more effective relative to a 5.0–6.0 µm wavelength, with the wavelength of 2.5–3.0 µm having shorter drying time relative to 5.0–6.0 µm. Similarly, Zhang et al. [118] reported that a decreased drying time was associated with a wavelength of 2–4 µm compared to the drying time for a 0.25–2 µm wavelength in combined medium- and short-wave IR and hot air drying of sponge gourd. It is important to note that the impact of IR wavelength on food processing and drying is codependent on factors such as temperature and moisture content of foods. Hamanaka et al. [114] investigated the inhibition susceptibility of *Bacillus subtilis* at a water activity of 0.7 when treated with three different wavelengths of IR heaters (950, 1100, and 1150 nm). The results showed that the inhibition of pathogenic microorganisms at 950 nm was higher than at other wavelengths at the same temperature, thus highlighting that the efficiency of microbial inhibition and inactivation was affected by the radiation wavelength.

### 6.5. Food Depth 

Slice thickness has been investigated in respect to the effect of IR treatment, and it has been proven that food depth, i.e., thickness or thinness of food materials, affects IR drying significantly, regardless of IR intensity [118]. Wu et al. [119] investigated the effect of varying thicknesses (0.6, 0.8, 1.0, 1.3 mm) on non-fried potato chips subjected to IR treatment. Results showed that thin slices had faster rates of drying and enzyme inactivation relative to thicker slices. This result agrees with a previous study [120], which evaluated the thermodynamics and quality performance of drying kiwi slices (4 and 8 mm) in hybrid hot air-IR drying with ultrasound pretreatment, reporting that a greater amount of thermal efficiency was recorded for the thinner kiwi slices of 4 mm.

## 7. Application of Hurdle Technology and Infrared in Food Processing/Preservation

The hurdle concept, which combines several preservative methods, often with reduced intensities, has emerged as a promising approach for food preservation that reduces nutritional and sensory quality losses while also improving food safety [121]. Combining hurdles such as osmotic drying, infrared drying, high-density polyethylene packaging, and irradiation has been utilized to elongate the shelf life of different foods over the years. These have been employed for fruits such as pineapple slices in ambient storage [122] and green peas using NIR drying and other technologies [123]. Infrared heating can be used alongside other technologies for food preservation, mainly because of its ease of modification, adaptability, and simplicity of the equipment. It can easily be used in combination with other heating methods such as convective, vacuum, and microwave heating [36]. Combining infrared radiation with other drying methods as a hurdle technology is very promising because it improves the drying rate and preserves food quality [36].

In combination with hot air drying, infrared heating has been used in processing rice [109], paddy [124], longan [106], murta berries [125], sweet potatoes [126], sponge gourd [118], green peas [123], and kiwifruit [120]. It has also been used in combination with vacuum drying in processing pomegranate arils [127], potato slices [128], goji [129], lemons and grapefruits [130], pumpkin [107], and button mushrooms [130]. Microwave drying and IR drying have been employed in drying raspberries and green peppers [131], while freeze-drying and infrared have been used in conjugation for the treatment of mushrooms [132] and chives [133]. Si et al. [112] reported the combined drying kinetics of IR and varying microwave powers (400 600, 800 W) and vacuum pressures (45, 65, 85 kPa) on fresh raspberries. Results obtained from this study showed reduction in drying time by half relative to IR drying only and twice the crispiness value. Similarly, in other studies, IR and heat pump drying was used in preserving Chinese yams [108] and grated carrots [134] with varying effects. Bualuang et al. [109] reported the drying kinetics and adequacy in nutrition of parboiled rice using a variety of drying methods including hot air, IR (1000 W and 1500 W; 60–100 °C), and a combination of both methods. IR showed relatively low energy consumption and reduced drying time due to high penetrability of IR waves that facilitate rapid heating processes. The combination of IR with other novel food processing techniques improves food preservation and quality while reducing processing time, energy requirements, and costs. Table 1 highlights the application of IR and other treatments in food processing and preservation.

## 8. Advantages and Disadvantages of Infrared Heating

IR technology is energy-efficient, with features such as heating homogeneity, high heat transfer rate, quick heating, low energy consumption, and improved product quality, making it a desirable food processing technology. Furthermore, IR technology is considered an environmentally friendly heating technique, primarily because IR heating does not require a heating liquid medium; hence, it is a water-saving technique. Additionally, IR heating is a non-contact, chemical-free method of disinfection, and this is of significant benefit in food processing, as it improves the quality of the treated products and reduces chemical and water consumption while also increasing manufacturing efficiency and food safety [22]. Other advantages include low energy costs, the small size of IR equipment, and the ability to precisely control the processes. Traditional heaters are less expensive to operate but provide less effective heating, which makes IR heating cost-effective. IR heaters generate energy that is focused directly on the material to be heated, with no volatile organic compounds, carbon monoxide, or nitrogen oxides produced. These properties of IR radiation have a direct positive impact on the organoleptic quality of foods.

However, IR radiation has some disadvantages, especially because of its high energy, which poses a risk of burns on foods due to improper application. Furthermore, its relatively small penetration depth in food is a challenge for its use in most food processing; thus the size of food products should be carefully considered when using IR heating [2]. Importantly, while IR radiation is relatively advantageous in the heating of solid food, it is not equally applicable in the treatment of most liquid foods [135], thus limiting its applicability. The limitation of penetration depth is easily accounted for in solid food, where heat from the surface spreads across the food via conduction. However, in liquid foods, heat distribution is relatively inefficient, especially in clear water-based food where particles are farther apart. Another potential limitation of IR heating is the time requirement, which is more than it is for other novel food processing methods such as microwave heat treatment. In a comparative study of IR and microwave heating on paprika powder, Eliasson et al. [32] reported a longer IR heating time (3.7 min) relative to microwave heating (1.5 min). This was speculated to be due to sample thickness and the limitation of IR penetration depth. Several other studies have reported a similar trend. Erdoğdu et al. [30] attributed IR longer heating time to the need to reduce IR heat flux in a stepwise manner to reduce possibility of surface overheating, while Borda-Yepes et al. [136] reported a higher rate of moisture removal from blueberry leaves using microwave heating relative to IR heating. The authors attributed this result to the difference in the heating mechanism. Importantly, it is difficult to compare findings from different studies due to the difference in power utilized and the fact that many studies employ a hurdle approach, combining several technologies to achieve thermal treatment or drying. Nevertheless, these limitations of IR are only minor disadvantages, as desired IR treatment of foods usually results in a relatively shorter duration compared to more conventional heating methods [137]. 

Interestingly, with the use of IR-based systems, the cleaning and maintenance of their components can be a challenge. IR heating banks are composed of individual electrical element cells housed within a ceramic or similar insulating-type block, which may make accessibility and handling difficult. Overall, this could easily lead to damage during the cleaning process relative to microwave heating systems, which are usually comprised of fewer complex parts [138]. In addition, the components of a typical microwave heating system are not exposed, which reduces the risk of damage, coupled with the fact that some microwave heating systems produce steam when heating food, which makes it easier to clean residues [139]. Similarly to this, for hot air heating systems with fixed inaccessible components, the risk of damage is equally reduced due to the inaccessibility of the components built in within the system’s cabinet [140]. Overall, IR heating technology offers numerous benefits in the food industry, but certain challenges as highlighted limit its use on different foods. Despite these drawbacks, IR heating remains a promising and increasingly viable option for sustainable food processing.

## 9. Conclusions

Food products are thermally treated for the purpose of extending their shelf life, ensuring food safety, and/or improving flavor and taste. One of the major factors affecting the profitability of many food processing operations is energy conservation. Several food processing operations, including drying, baking, roasting, blanching, pasteurization, and sterilization, can be accomplished using novel infrared radiation heating technology. IR radiation, which falls within the electromagnetic spectrum within the borders of visible light and microwaves, is a green thermal emerging technology that can further be exploited in food processing due to the advantages it offers to meet current consumer needs and trends within the food industry relative to existing methods. IR heating has several advantages over conventional methods such as convection heating due to its higher energy efficiency, low energy consumption, and improved product quality. In the food industry, IR energy has the potential to be used to process high-value and value-added food products such as fruits, vegetables, spices, juices, and other biomaterials with positive impacts on the physiochemical properties and sensory characteristics of food components, which further justify its use in the food industry. This review discussed and illustrated the use of IR technology in various aspects of the food manufacturing process. It further highlights the application of hurdle technology and IR in food processing/preservation as well as factors affecting the efficiency of the method to guide a holistic understanding of its application. Nonetheless, the use and understanding of IR heating in food processing and microbial decontamination of food is still evolving. 

IR heating can be combined with other conventional and emerging technologies to improve its applicability as well as reducing the limitations of existing combined methods. Combining IR heating with microwave heating and other common conductive and convective modes of heating has a lot of potential for achieving energy efficiency and practical applicability in the food processing industry. To maximize the benefits of IR heating, further research is essential to standardize processes and address its limitations, particularly regarding penetration depth. Furthermore, there is a need for a thorough understanding of the theoretical underpinnings of IR radiation heating effects, particularly as they relate to food composition, changes to molecules that alter organoleptic attributes, taste, and flavor, and the mechanisms of enzymes, bacteria, and fungi inactivation during food processing. Collaborative efforts among researchers and industry stakeholders are crucial to developing and implementing these innovative technologies effectively. Ultimately, the adoption of IR heating not only holds promise for improving food processing efficiency but also has broader implications for food safety, waste reduction, and global food security.

## Figures and Tables

**Table 1:** Application of hurdle technology and IR in food processing/preservation.

Hurdle Technology	Food Type	Parameters	Observation	References
Hot air (HA) and IR drying	Rice	IR intensity: 1000 and 1500 W, air flow rate: 1.0 ± 0.2 m/s at 30 °C.	Relatively efficient drying kinetics while maintaining chemical quality and whiteness of rice.	[109]
	Paddy	IR intensity: 2000 W/m^2^, inlet air velocity: 0.15 m/s at 30 °C	Improved sensory quality of paddy, which is a heat-sensitive product with a moderate specific energy consumption (SEC).	[124]
	Kiwifruit	Ultrasonic pre-treatment, hybrid hot air-IR dryer at 500 W, temperature levels of 50, 60, and 70 °C	Increased drying efficiency, thermal efficiency, and energy efficiency. Decreased SEC, shrinkage, and drying time.	[120]
	Murta Berries	Drying under combined convective-IR at 40, 50, and 60 °C. IR intensity: 400–800 W.	Increased IR power increased the retention of total phenolic content (TPC) in sample. Dried samples with the highest TPC were obtained at 40 °C /800 W.	[125]
	Sweet Potatoes	Ultrasonic pre-treatment at 20, 40, 60 kHz, 30 min, 300 W/cm^3^. HA temperature: 60,70, and 80 °C. Air velocity: 1.5 m/s	Energy efficiency with high retention of food quality. Ultrasonic pretreatment at 40 kHz combined with IR and HA (70 °C) significantly reduced drying time.	[126]
	Green Peas	Drying air temperature of 30, 40, and 50 °C. IR intensity levels of 2000, 4000, and 9000 W/m^2^	Decreased moisture content from 75.34 ± 0.53 to 20.02 ± 0.14 (% w.b) at higher drying rate. Optimum condition was 50 °C and 4000 W/m^2^	[123]
	Kiwifruit	Ultrasonic pre-treatment, hybrid hot air-IR dryer at 500 W, temperature levels of 50, 60, and 70 °C	Increased drying, thermal, and energy efficiency. Decreased SEC, shrinkage, and drying time.	[120]
	Longan	FIR rods powered at 250, 350, and 450 W, FIR-hot air (65 °C) with 80% recycled air.	Initial moisture content of ~86% w.b was reduced to 18% w.b at a relatively shortened drying time and maintained sensory qualities of longan.	[106]
Vacuum and IR drying	Pumpkin	Vacuum pressure: 5–15 kPa, time: 0–220 min. IR power: 204–272 W	Optimally dried pumpkin slices with maximum retention of β-carotene were obtained at 238 W and 5 kPa. However, high IR power caused color change.	[107]
	Lemon slices	Vacuum pressure: 5, 15, and 25 kPa, time: 0–140 min. IR power: 300, 350, and 400 W	Decreased moisture content and increased drying rate were observed with increased power and observable color difference.	[130]
	Goji/Wolfberry	Vacuum pressure: 3.0 kPa and time: 10, 15, and 20 min. Drying temp: 60, 65, and 70 °C. IR power: 250 W	Relative to hot air drying, combined vacuum, and IR treatment at 65 °C, vacuum pressure duration of 15 min and ambient pressure of 2 min enhanced drying and quality of dried food without chemical pretreatment.	[129]
	Potato Slices	IR power: 100, 150, and 200 W. Vacuum pressure: 20, 80, and 140 mmHg.	Reduced drying time, low shrinkage, and high rehydration capacity occurred with increased IR power, moderate vacuum level, and thin slice thickness.	[128]
	Pomegranate Arils	NIR-vacuum dryer. Absolute pressures: 2, 20, 40, and 60 kPa. Drying temperatures: 60, 70 , 80, and 90 °C.	Minimum shrinkage and color alteration were observed at temperature of 60 °C and absolute pressure of 2 kPa with an increasing efficient mass transfer rate.	[127]
Microwave and IR-HA drying	Green Pepper	HA drying temperature: 65 °C. Air velocity: 1.8 ± 0.1 m/s. Microwave power: 62 W. IR power: 240 W	Combined methods influence drying kinetics, color retention, water activity, and rehydration positively.	[131]
IR and Freeze-drying	Chives	Freeze-drying temperature: −80 °C using a catalytic IR dryer.	Combined method is comparatively suitable relative to drying time and quality of product.	[133]
	Mushroom	IR intensity: 5.8 kW/m^2.^. Pressure: 100 Pa. Cold temperature: −35–(−40) °C.	Increased drying rate. Significant aroma retention and increased sulphur compounds in food.	[132]
IR and microwave-vacuum drying	Raspberries	Microwave powers: 400, 600, 800 W and vacuum pressures: 45, 65, 85 kPa	Reduction in drying time by half relative to IR drying only and twice the crispiness value.	[112]
IR and heat pump drying	Chinese Yam	Heat pump dryer of 1.5 kW. IR intensity: 500, 1000, and 2000 W (500, 1000, and 2000) FIR, respectively. Air velocity: 1.0 m/s at 50 °C	Combined methods showed high values of crispiness and relatively greater coloration compared to heat pump dried samples. Best products were obtained at 1000 FIR.	[108]
	Grated Carrot	Temperature: 45 and 50 °C Air velocity: 0.5 m/s IR lamp: 2.5 µm wavelength	Energy efficiency was observed with combined methods. Moisture content reduced from 7.06 g water/g dry matter to 0.14 g water/g dry matter.	[134]

## Data Availability

No new data were created or analyzed in this study. Data sharing is not applicable to this article.
